# Network-Based Single-Cell RNA-Seq Data Imputation Enhances Cell Type Identification

**DOI:** 10.3390/genes11040377

**Published:** 2020-03-31

**Authors:** Maryam Zand, Jianhua Ruan

**Affiliations:** 1 Department of Computer Science, University of Texas at San Antonio, San Antonio, TX 78249, USA; 2 Department of Molecular Medicine, University of Texas Health Science Center at San Antonio, San Antonio, TX 78229, USA

**Keywords:** scRNA-seq data, data imputation, co-expression network, graph random walk, clustering

## Abstract

Single-cell RNA sequencing is a powerful technology for obtaining transcriptomes at single-cell resolutions. However, it suffers from dropout events (i.e., excess zero counts) since only a small fraction of transcripts get sequenced in each cell during the sequencing process. This inherent sparsity of expression profiles hinders further characterizations at cell/gene-level such as cell type identification and downstream analysis. To alleviate this dropout issue we introduce a network-based method, netImpute, by leveraging the hidden information in gene co-expression networks to recover real signals. netImpute employs Random Walk with Restart (RWR) to adjust the gene expression level in a given cell by borrowing information from its neighbors in a gene co-expression network. Performance evaluation and comparison with existing tools on simulated data and seven real datasets show that netImpute substantially enhances clustering accuracy and data visualization clarity, thanks to its effective treatment of dropouts. While the idea of netImpute is general and can be applied with other types of networks such as cell co-expression network or protein–protein interaction (PPI) network, evaluation results show that gene co-expression network is consistently more beneficial, presumably because PPI network usually lacks cell type context, while cell co-expression network can cause information loss for rare cell types. Evaluation results on several biological datasets show that netImpute can more effectively recover missing transcripts in scRNA-seq data and enhance the identification and visualization of heterogeneous cell types than existing methods.

## 1. Introduction

In the past decade, advancements in next-generation sequencing technologies have revealed unprecedented insights into complex biological systems. Recently, these types of technologies have been increasingly developed to scrutinize a diverse range of phenomena at single-cell resolutions. In contrast to the traditional profiling methods that examine bulk populations, these single-cell analyses have enabled researchers to achieve novel breakthroughs in biological problems [1,2,3,4]. For instance, single-cell RNA sequencing (scRNA-seq) has been proven effective in revealing cell type heterogeneity and developmental trajectories along with uncovering gene regulatory network [5,6].

The recently emerging single-cell transcriptomic technologies each have their own unique competencies accompanied by weaknesses and restrictions regarding their accuracy, sensitivity, throughput, and precision. They suffer from technical noises mainly rooted in the low amount of starting mRNA in each cell. This causes as small as 5–15% of transcriptomes to be captured throughout the amplification process, leading to a partially observed version of the actual expression profile [7,8,9]. For genes with a low level of expression, this will result in the appearance of excess incidence of zeros in the expression count matrix, often referred to as “dropout event.” This inherent sparsity of scRNA-seq datasets hinders further characterizations at cell/gene-level such as cell type identification and downstream analysis. More specifically, “general-purpose” clustering algorithms often fail to distinguish rare cell types because the aforementioned sparsity can provide a false view of expression level and may invalidate their similarity measurements. It is worth mentioning that improvements are made in this area by adopting heavier models with the cost of demanding more computational power and effort to implement [10,11,12,13,14,15,16].

To overcome the challenges associated with scRNA-seq data, several computational methods have been proposed, centered around the idea of data imputation. To name a few, MAGIC (Markov Affinity-based Graph Imputation of Cells) [17], scImpute [18], SAVER (Single-cell Analysis Via Expression Recovery) [19], and netSmooth [20] have become popular tools that generally impute missing read counts using observed transcripts. More specifically, MAGIC attempts to recover real gene expression values by sharing information across similar cells, based on the concept of heat diffusion, which is conceptually similar to the well-known Personalized Page-Rank method [21]. The scImpute assumes that most genes have a bimodal expression pattern that can be described by a mixture model with two components. The first component is a gamma distribution used to account for the dropouts, while the second component is a normal distribution to represent the actual gene expression levels. It then estimates missing values by borrowing information from the values of other similar cells for the same gene. SAVER is a Bayesian-based method that recovers the real expression for each gene in each cell by a weighted average of the observed count and the predicted value. The predicted value is estimated by the observed expression of some informative genes in the same cell. SAVER assumes that the count of each gene in each cell follows a Poisson-gamma mixture, also known as a negative binomial model. On the other hand, netSmooth is a network diffusion-based method that uses prior knowledge (PPI network) to smooth the expression count matrix.

Although these imputation methods have partially helped alleviate scRNA-seq data sparsity, each of them is bound by some unique limitations. For instance, MAGIC is prone to over-smoothing the data and inducing spurious correlation among genes. Its performance is also very sensitive to the choice of diffusion time, as in theory when diffusion time approaches infinity the smoothing kernel can reach a stable distribution regardless of the starting distribution. SAVER replaces missing values with near-zero values which limits its ability to help with clustering. The netSmooth makes explicit use of the PPI network to estimate missing values although the available PPI network is usually static and lacks cell type context. The performance of scImpute on real data is not as good as it is on simulated data. It also asks for cell labels or number of clusters as a priori input, which is not readily available for most datasets. Finally, some tools such as scImpute and SAVER heavily rely on specific statistical distribution assumptions, which may lack generality/efficiency as noise distributions are often unknown and may be subject to change with the introduction of new experimental technologies for single-cell transcriptomics.

In this study, we introduce netImpute, a network-based approach that imputes a zero-inflated raw dataset by diffusing transcript read counts on the gene co-expression graph through the application of a Random Walk with Restart (RWR) process. The low count genes whose neighbors are relatively highly expressed (deemed as potential dropout candidates) are smoothed based on a weighted average of their adjacent genes in the co-expression network. While the netImpute algorithm is similar to netSmooth and MAGIC in spirit, they differ in the network choices and/or diffusion process, which in our view are ultimately responsible for the performance difference between the different algorithms, as will be demonstrated on several scRNA-seq datasets in this study.

## 2. Materials And Methods

### 2.1. Datasets

#### 2.1.1. Simulation Study

We generate four different simulation datasets, each corresponding to different levels of dropout severity, following a similar procedure proposed in scImpute. We consider 150 cells divided into three different cell types c1, c2, and c3 based on their 270 highly expressed genes out of the total of 20,000 genes. We generate the so-called “original data” and “raw data” where the former encompasses initial gene expression values and the latter is filled by excessive zeros representing dropouts. We create the original data which is directly log-transformed in two steps (i) mean and standard deviations of expressions for all genes are obtained from two Normal distributions with mean 1.8 and standard deviation 0.5 and mean 0.6 and standard deviation 0.1, respectively; (ii) for each cell type we randomly select 270 genes and multiply their mean expressions by a random number in range from 2 to 10. We do this to mimic the reality in which only about 810 genes are truly differentially expressed. Eventually, we arrive at our raw data by injecting extra zeros to the original data based on a Bernoulli distribution with a dropout rate following a double exponential function $exp(-\lambda *meanExp^{2})$ (meanExp is the mean expression of a given gene), as assumed in [22]. We then build four different raw datasets with dropout rates of 72,77,82,87% corresponding to different values of λ (0.1, 0.08, 0.06, 0.04).

#### 2.1.2. Biological Datasets

In this study, we apply netImpute and four other methods (MAGIC, scImpute, netSmooth, and SAVER) on seven publicly available scRNA-seq datasets [23,24,25,26,27,28,29]. We choose these datasets because they cover a realistically wide range of possible characteristics of experimental datasets such as the number of cells and cell types. More specifically, they have a different number of cells ranging from 80 to 8000. The number of clusters varies from four to 14 based on what is reported in original publications. These datasets are shown in Table 1. Investigating such a rich selection of datasets largely enables us to examine the performance of our proposed method as well as other available tools. In netImpute, we also use the PPI network to evaluate its performance against using of gene-gene or cell-cell networks. We obtain this PPI dataset from string-db [30]. Interactions are subsequently filtered to only keep the 10% most confident ones (based on combined interaction score). This led to a network with 17,013 genes and 1,020,816 interactions for mouse and a network with 852,722 interactions among 17,467 genes for humans.

### 2.2. Data Processing and Normalization

It is known that scRNA-seq data usually have a high level of cell to cell variation in library size (number of observed molecules). This variation is largely due to technical noise rather than biological reasons [8]. For example, cell barcode can have a significant effect on the PCR efficiency and the number of transcripts in that cell. Therefore, we need to normalize data based on library size, so that each cell will have the same count number. Normalized count matrix is calculated as follows: each read count from the expression matrix is divided by the total reads in that cell, then multiplied by the median of total read counts across all cells so that all cells have an equal number of reads. Formally, given expression matrix $E_{m*n}$ the normalized data matrix is defined as

$$E_{norm}\left(i,j\right)=\frac{E(i,j)}{\sum_{k=1}^{m}E\left(k,j\right)}*median\left(libSize\right)$$

where, libSize=colSum(E) and *m* in the number of genes and *n* is number of cells. In the end, we apply a log transformation with a pseudo count of 1. The pseudo count is added to avoid infinite values. The benefit of the logarithmic transformation is to reduce the influence of a few large observations in data.

### 2.3. Overview of Netimpute Algorithm

The main intuition behind netImpute is to see if diffusing the gene expression network by borrowing information from neighboring genes can smooth scRNA-seq data and improve cell type identification and visualization. The driving motivation here is that when the expression level of a given gene is recorded very low (almost zero) in the scRNA-seq process while its local neighbors are highly expressed, it is presumable that it might have been a technical noise that has led to the zero count, rather than it being a real silent gene. In this scenario, netImpute attempts to recover dropouts by mimicking the concept of data diffusion. The data diffusion process shares information among genes through local neighbors to impute the gene expression and discover the manifold structure. Data diffusion has led to promising results in finding trajectories and dealing with dropout events of RNA sequencing in single-cell data [17,31,32]. netImpute takes as its only input the gene expression matrix, *m* genes by *n* cells, and then calculates the Pearson correlation among the genes to construct the gene similarity matrix. The mutual k-nearest-neighbor (KNN) algorithm is then applied to this gene similarity matrix to build the co-expression network. In this network, nodes denote genes and links connect each gene to a neighborhood of its most similar genes. This network is then diffused through implementing an RWR, which results in a probability matrix representing the influence of each gene on the others. In the next step, the transpose of this probability matrix is multiplied with the initial noisy expression matrix. This process of generating post-smoothed data effectively estimates more realistic activity level of a gene-based on the read counts of that gene as well as its neighbor genes. Thus, a gene with a low read count (a possible dropout) whose neighbors have high expression level will get an increase in its expression level given by its neighbors. We discuss the details of different steps of netImpute in the following sub-sections.

#### 2.3.1. Construction of the Gene Co-Expression Network

netImpute starts with an m*n count matrix *E*, representing the observed read counts of *m* genes in *n* cells. To compute similarities between gene vectors in the count matrix *E*, netImpute calculates Pearson correlation coefficient between all gene pairs which leads to the gene-similarity matrix *S*. Next, it generates co-expression matrix *A* by applying mutual KNN algorithm on matrix *S*. This network represents a faithful neighborhood of genes derived from a decent similarity metric based on the expression patterns. More precisely, for each gene $G_{i}$ in *S*, let $N_{k}\left(G_{i}\right)$ be the *k* nearest neighbors of $G_{i}$, co-expression graph is defined as follows: two genes are considered co-expressed if both of them are in the top-*k* neighborhood of each other. Formally, $G_{i}$ and Gj are connected if:

$$G_{i}\in N_{k}\left(G_{j}\right)\;AND\;G_{j}\in N_{k}\left(G_{i}\right)$$

Another alternative method to construct *A* is asymmetric KNN [33], where two genes are related if either of them are in the top-*k* neighborhood of the other. Formally, $G_{i}$ and Gj are connected if:

$$G_{i}\in N_{k}\left(G_{j}\right)\;OR\;G_{j}\in N_{k}\left(G_{i}\right)$$

We prefer to use mutual KNN over asymmetric KNN mainly because the former appears to predict more reliable links between genes, giving a less noisy network. As adding false positive signals to the data during imputation is a big concern in the field, we alleviate this issue by constructing a network with high-quality links only.

Currently, it is still under debate that borrowing information from which source (i.e., genes, cells, or an external source such as PPI) is a more effective approach to handle dropouts. Even though the main focus of netImpute is on using genes, for the sake of completeness and flexibility we also studied the cases where we replace the abovementioned gene co-expression network with cell co-expression network or PPI. To obtain the cell co-expression network, the same procedure is followed as that of gene-gene network. The PPI network is retrieved from [30] and is filtered out to only keep 10% high confident interactions.

#### 2.3.2. The Random Walk with Restart (RWR) Process

The netImpute algorithm receives the constructed co-expression network *A* where nodes are genes and edges between them implies that they are co-expressed. The aim of this step is to walk through *A* and build a probability matrix that gives an estimation of the influence of each gene on the other genes. Mathematically speaking, let *A* be the adjacency matrix of an unweighted, undirected graph, where $A_{ij}=1$ if there is an edge between node *i* and node *j* and 0 otherwise, and *P* be the row normalized adjacency matrix defined on the graph, where $p_{ij}=\frac{A_{ij}}{\sum_{j}A_{ij}}$ is the transition probability from node *i* to node *j*. Assume that a random walker starts from a node *v*, with a uniform probability to visit each of its neighboring nodes, and with a fixed probability α to revisit the starting node *v* at any time point during the walk. The extent of how the expression level of a gene is affected by its neighbors largely depends on α; low value for α increases this influence while the high value for α decreases it leading to more local diffusion. A value between 0.3 to 0.7 usually provides the best performance for the datasets used in this study. The probability for the random walker started at node *v* to be present at any node *j*, at a discrete time point *k*, is

$$f_{vj}^{\left(k\right)}=\left(1-\alpha \right)\sum_{i}f_{vi}^{\left(k-1\right)}p_{ij}+\alpha f_{vj}^{\left(0\right)}$$

where, $f_{vj}^{\left(0\right)}=1$ if v=j, and 0 otherwise. This can be computed using the matrix form

$$F^{\left(k\right)}=\left(1-\alpha \right)F^{\left(k-1\right)}P+\alpha I$$

where, *F* is a square matrix and I is an identity matrix.

This random walk procedure is known to be convergent [34] and the stationary probability $f_{vj}^{\left(\infty \right)}$, or simply $f_{vj}$ for short, is the probability for a random walker that started at node *v* to reach node *j* at equilibrium.

Having this probability matrix in hand, netImpute generates its main output of imputed data by adjusting the gene expression level of each gene via a weighted average of its own expression value and its neighbors’ expression values. Formally, let $e_{vc}$ be the observed expression value of gene *v* in cell *c*, then the imputed value is calculated by the following equation:

$$e_{vc}^{imputed}=\sum_{j}f_{vj}e_{jc}=f_{vv}e_{vc}+\sum_{j\neq v}f_{vj}e_{jc}$$

As $\sum_{j}f_{vj}=1$, and $f_{vv}\geq \alpha$ for any *v* due to the probability to revisit the starting node, it is easy to see that $\sum_{j\neq v}f_{vj}\leq 1-\alpha$. Therefore, the parameter α determines the relative contribution of a gene’s own expression level and its neighbors’ expression levels. One notable difference between netImpute and an existing method, netSmooth, lies in the fact that netSmooth uses incoming probabilities, i.e., $f_{jv}$, while netImpute uses outgoing probabilities $f_{vj}$. Due to this difference, netImpute ensures that the imputed expression level for each gene has a similar magnitude as in the original data independent of its number of neighbors in the network, while the imputed value for a gene from netSmooth is significantly biased by its network degree, as will be demonstrated and discussed in Results.

### 2.4. The Evaluation Measure for Clustering

We use two clustering algorithms PCA+kmeans and SC3 to assess the effectiveness of data imputation in the improvement of cell-type identification. SC3 [13] is a kmeans-based ensemble clustering method that first calculates a cell distance matrix, followed by various matrix transformations and kmeans clustering. Instead of optimizing parameters (e.g., distance metric, matrix transformation), SC3 combines several clustering outcomes and outputs an averaged result. The number of clusters is given as a priori for both clustering methods. A recent large-scale evaluation study shows that SC3 has the best overall clustering performance [35]. Another popular tool, Seurat [11], has similar performance as SC3, which is also confirmed in our study; however, Seurat is a much more complex workflow for comprehensive single-cell data analysis, including normalization and imputation, and therefore is less suitable than SC3 for independent evaluation of the impact of imputation on clustering [36].

To compare the clustering performance of netImpute against other existing imputation tools, we compute and analyze the adjusted rand index (ARI) of the clustering results compared to the gold standard [37]. The gold standard used in this study is the cell labels reported in the original publication. The cell types either come from experiments that include cells from different stages, conditions, and lines (i.e., Pollen, Deng) or were identified through some computational methods (i.e., Treutlein, Zeisel, Usoskin). Given a set of objects $S=s_{1},s_{2},\ldots ,s_{n}$, let $X=X_{1},X_{2},\ldots ,X_{M}$ and $Y=Y_{1},Y_{2},\ldots ,Y_{N}$ represent the true and predicted partitions of the objects, where each object appears in *X* and *Y* exactly once. Let $n_{ij}$ be the number of common objects between $X_{i}$ and $Y_{j}$. The ARI can then be calculated as

(1) ARI=∑ijnij2︷Index−[∑ini•2∑jnj•2]/n2︷ExpectedIndex12[∑ini•2+∑jnj•2]︸MaxIndex−[∑ini•2∑jnj•2]/n2︸ExpectedIndex

where $n_{i•}=\sum_{j}n_{ij}=\left|X_{i}\right|$ is the size of $X_{i}$, and $n_{j•}=\sum_{i}n_{ij}=\left|Y_{j}\right|$ is the size of $Y_{j}$. The ARI penalizes both false positive and false negative decisions.

### 2.5. Benchmarking

We compare netImpute against several imputation tools including MAGIC, scImpute, netSmooth, and SAVER. We apply clustering techniques PCA+kmeans and SC3 on raw and imputed data. Then, we evaluate the clustering accuracy obtained through netImpute and that of those tools. We examine seven different scRNA-seq datasets present in Table 1 along with their major characteristics such as expression units, number of cells/clusters, etc. To feed these datasets into netImpute and the other four imputation tools we first remove “low quality” genes defined as those genes who are not expressed in any cell. Whether we perform data normalization or not depends on the specifics of the imputation tool which we explain in the following for all individual tools.

MAGIC: We download MAGIC 1.4.0 form GitHub (https://github.com/KrishnaswamyLab/MAGIC). For MAGIC prior to imputation data should be library size normalized explained in the Methods section of this paper. After normalization, we transform data using a square root function as opposed to the commonly used log transformation following the recommendation of the MAGIC authors in their tutorial. This was done to alleviate the instabilities at zero which would otherwise arise if log transformation was performed. MAGIC has several input parameters such as diffusion time (t = ‘auto’) and the number of nearest neighbors on which to build the kernel (KNN = 10) which were all set to their default values.

scImpute: We download scImpute 0.0.7 form GitHub (https://github.com/Vivianstats/scImpute). In accordance with their tutorial, no normalization is needed on raw data. Instead, normalization is taking care of as an internal step of the tool itself. The number of cell clusters (Kcluster parameter) which is a priori variable required by scImpute is set equal to the expected number of cell clusters in each dataset reported in Table 1. All other parameters are left to their default values.

SAVER: We download SAVER 1.1.1 from GitHub (https://github.com/mohuangx/SAVER). Similar to scImpute no normalization is done on raw data. All the parameters are set to their default values.

netSmooth: We download netSmooth 1.4.0 from GitHub (https://github.com/BIMSBbioinfo/netSmooth.git). The input data has been log normalized. Parameter alpha is set to 0.5. All other parameters are set to their default values.

PCA+kmeans: Here PCA and kmeans functions from the scikit-learn python package are used for clustering. We apply PCA on imputed data for dimension reduction where a consistently equal number of principal components (PCs) is used for all the datasets (n_components = 20). Kmeans parameter of the maximum number of iteration and number of times that it will be run with different centroid seeds are set to 1000 and 100, respectively ( max_iter = 1000, n_init=100).

SC3: We download SC3 1.10.1 form R Bioconductor (http://bioconductor.org/packages/release/bioc/html/SC3.html). To ensure the consistency of SC3 for all methods gene filtering option is turned off (gene.filter = False) and the number of cell clusters (ks) is set to the expected number of cell clusters in each dataset reported in Table 1.

Software is written in python and it can be downloaded from http://www.cs.utsa.edu/~software/netImpute/.

## 3. Results

### 3.1. Performance Evaluation of Netimpute on Simulated Data

**Figure 1:**
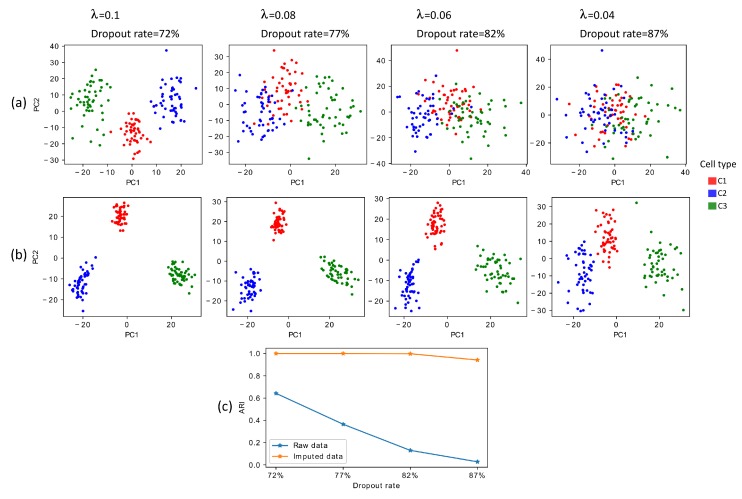
(Color online) netImpute improves data visualization and clustering performance on simulated data. (**a**) The first two dimensions calculated from the raw datasets with different dropout rate; (**b**) the first two PCs calculated from imputed datasets by netImpute; (**c**) clustering accuracy for both raw and imputed datasets using PCA+kmeans clustering method.

As the first step to assess the performance of our method, we design a simulation study in which we generate gene expression data for 150 cells divided into three cell types, with a total of 20,000 genes, out of which 810 genes are differentially expressed (for more details, refer to Methods). We systematically increase the proportion of zeros (i.e., average dropout rate) from 72% to 87% to mimic different levels of complexity arising from excess zeros. This selected range for the dropout rate is within that of observed in real biological datasets [23,26,27]. Then, we apply the Principal Component Analysis (PCA) to raw data (Figure 1a) and netImpute-treated data (Figure 1b).

Visualization of simulated data imputed by netImpute demonstrates that netImpute can easily separate cell types with just one dimension. This enhancement remains present even for intensely imputed data with the dropout rate as high as 87%.

To display the performance of netImpute in a more quantitative way, we apply kmeans clustering to PCA reduced data for both raw and netImpute-treated data. Figure 1c shows that the Adjusted Rand Index (ARI) remains constantly near unity for netImpute-treated data, whereas it continuously diminishes for raw data and approaches zero as dropout rate increases to 87%. Therefore, PCA visualization and PCA+kmeans clustering both indicate that netImpute can effectively detect different cell types by recovering missing values in data severely affected by dropouts.

### 3.2. Performance Evaluation of Netimpute on Real Data Using Different Types of Networks

To evaluate the performance of netImpute on improving cell type identification from real scRNA-seq data, we apply netImpute to seven scRNA-seq datasets covering a wide range of experimental protocols, dropout rates, and cell numbers. PCA+kmeans is then used to cluster both the raw data and the imputed data. While netImpute is envisioned to work best with gene co-expression network, the general algorithm can be applied to other types of networks such as cell co-expression network or PPI network. As there is no available guideline in the literature about which type of network is most effective in scRNA-seq data imputation, here we evaluate and compare the performance of netImpute using all three types of networks for each dataset.

To eliminate evaluation bias that may be caused by parameter tuning, we treat each dataset with netImpute using a large range of parameter values (see Methods), and apply PCA+kmeans to the imputed data matrix resulted from each parameter setting. Adjusted Rand Index is then calculated corresponding to different values of the free parameters α (the restart probability of random walker) in range [0.1:0.9] in 0.1 increments and *k* (the number of neighbors in mutual KNN) in range given as $2^{i}$ for *i* from 3 to log2(m/4) where *m* is the number of genes.

**Figure 2:**
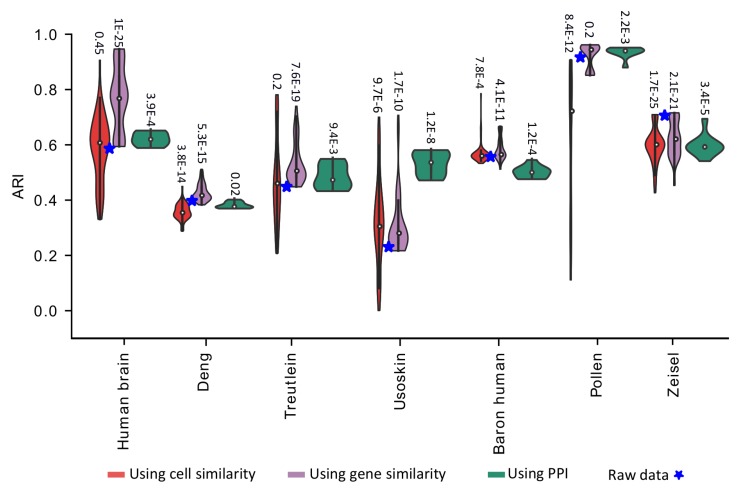
The netImpute utilizing information from gene similarity significantly enhances the clustering accuracy of real datasets.Violin plot showing the ARI obtained by netImpute-treated data based on cell similarity and gene similarity for different parameter settings (α and *k*) and based on the protein–protein interaction (PPI) network for different values of α. Values on top of the violin plots show the *p*-value obtained from *t*-test comparing the adjusted rand indices (ARIs) from imputed data with ARI from raw data.

As we can see from Figure 2, netImpute with gene co-expression network clearly improves clustering accuracy in five out of seven datasets, for almost all tested parameter values. While netImpute does not offer a substantial improvement in the Pollen and Zeisal datasets, it is noteworthy to mention that those datasets are already well-separated and have high clustering accuracy (ARI > 0.7) in raw data. Nevertheless, in these two datasets, netImpute does not appear to distort the raw data, thus maintaining their original structure. Another important observation is that generally better ARI is achieved with gene co-expression network compared to cell co-expression network or protein-protein interaction network. In addition, the results with gene co-expression network are far more stable and insensitive to values of α and *k* compared to cell co-expression network. This discrepancy is very pronounced for datasets having fewer number of cells, e.g., Treutlein with 80 cells, whereas it diminishes for datasets with a high number of cells such as the Baron human dataset with more than 8000 cells. This behavior is understandable, as the cell-cell similarity graph resulted from a very small number of cells may not cover the complete cell phenotype space and therefore may result in very crude imputation. Cell co-expression networks may be more beneficial when a large number of cells are available. Combining netImpute with protein-protein interaction network does not guarantee improved clustering accuracy, simply because the network is static and may not be relevant to the cell type of interest. On the other hand, given that PPI network usually does not deteriorate the clustering of the raw data, it can still be a useful source of information for scRNA-seq data imputation when a reliable gene or cell co-expression network cannot be obtained.

### 3.3. Comparison with Existing Imputation Methods

**Figure 3:**
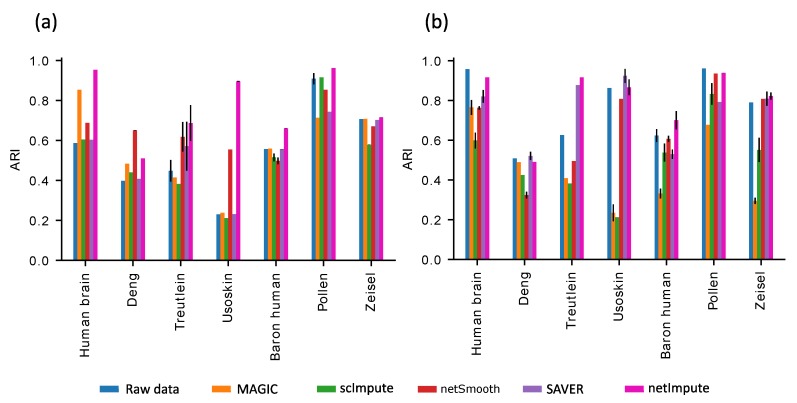
Clustering performance of data treated by netImpute compare to other existing methods. (**a**) Bar plot showing ARI obtained from applying PCA+kmeans on raw and imputed datasets (**b**) Bar plot showing ARI obtained from applying SC3 on raw and imputed datasets.

We apply two clustering methods, PCA+kmeans and SC3, on both raw and data processed by netImpute and other imputation tools including MAGIC, scImpute, netSmooth, and SAVER. Figure 3 shows the clustering accuracy assessed by ARI for all the above-mentioned cases. When PCA+kmeans clustering is used (Figure 3a), we can see that netImpute yields improved ARI values compared to that of raw data for all datasets and in several cases the improvement is dramatic. In addition, it outperforms all other imputation methods in every dataset except for one i.e., Deng. On the other hand, when SC3 is used, netImpute enhances the clustering accuracy only on half of the raw datasets (Figure 3b), but still surpasses other imputation methods in five out of seven datasets (Human brain, Treutlein, Baron human, Pollen, and Zeisel) and remains competitive for the other two. Our results are in line with [13], reinforcing the assertion that SC3 beats “general-purpose” clustering techniques (i.e., PCA+kmeans) in raw data cell identification. This roots in the fact that SC3 is a “specially-designed” clustering tool for processing scRNA-seq data by combining multiple clustering solutions through a consensus approach. Importantly, netImpute does not adversely disturb the structure of raw data thus maintaining the performance of SC3. In contrast, the other tools often degrade the performance of SC3. A detailed look into the confusion matrix obtained for one of the datasets, Treutlein, reveals that netImpute can differentiate Ciliated and Clara cell subpopulations, whereas PCA+kmeans or SC3 applied on raw data fails to do so (Table 2). In addition, netImpute helped SC3 to successfully separate AT1 cells from the rest.

**Figure 4:**
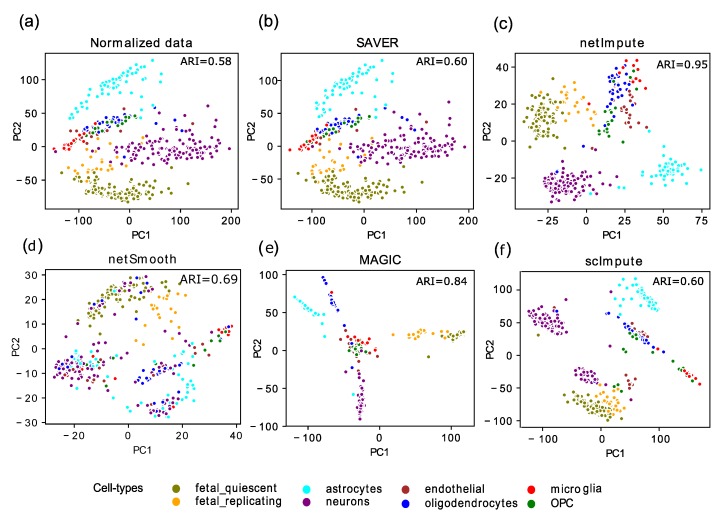
(Color online) Cell clustering and 2D PCA plot of human brain dataset. (**a**) no imputation; (**b**) after application of SAVER; (**c**) after application of netImpute; (**d**) after application of netSmooth; (**e**) after imputing with MAGIC; (**f**) after application of scImpute.

We further test netImpute to examine its performance in visually assisting with cell type identification task. In fact, many biologists prefer to have an initial feeling of gene expression data scattering in a simple 2D plot that helps with clustering and further analyses. Here we select the human brain dataset, apart from its significant biological importance, because it is a very noisy dataset in need of imputation. There are 420 cells in eight cell types in this dataset. Figure 4 shows the PCA plot of raw data and data imputed by netImpute, MAGIC, netSmooth, scImpute, and SAVER. We can see that the SAVER result is almost identical to the raw data. This is also quantitatively reflected in Figure 3a where ARI obtained by SAVER is very close to that of raw data. MAGIC seems to change data topology dramatically. The scImpute does not appear to improve the visualization and in some cases even divides a cluster into two sub-populations (e.g., neurons cell type). In the two-dimensional visualization by netImpute, the first PC largely separates fetal and adult neurons, while the second PC isolates neurons from other cells. This good performance of netImpute in identifying the members of each cell type is also reflected by achieving a high ARI value.

To evaluate the efficiency of imputation methods, we measured the run time of each method for the two largest datasets of this study (Baron and Zeisel). The most time-consuming part of our algorithm is in constructing the mkNN graph, which depends on the number of genes in the case of gene-gene co-expression networks. As shown in Table 3, the run time for SAVER and scImpute are almost impractical, for such modest-sized datasets nowadays. While MAGIC has the best run time, our method has a reasonable run time that is scalable to larger datasets. The shorter run time of netImpute on the larger Baron dataset than Zeisel is because the number of genes after filtering for Baron (17,000) is less than that of for Zeisel (20,000).

### 3.4. Impact of Different Diffusion Strategies

**Figure 5:**
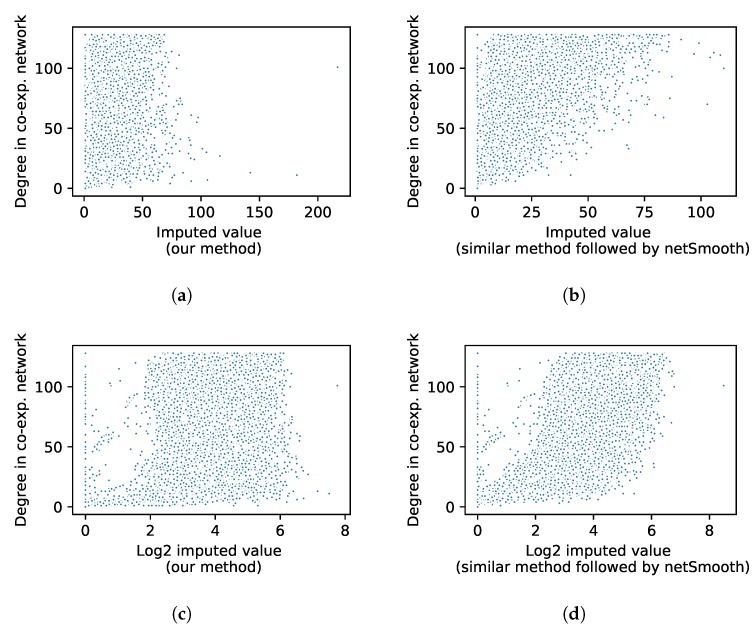
Scatter plot of gene’ degree in the network vs. its mean imputed values across all cells for netImpute (**a**) and netSmooth (**b**). Scatter plot of gene’ degree in the network vs. its log mean imputed values across all cells for netImpute (**c**) and netSmooth (**d**).

After applying RWR on the network, the probability for a random walker that started at node *v* to reach node *j* at equilibrium is $f_{vj}$, which is termed outgoing probability for node *v*. The summation of $f_{vj}$ for the outgoing probabilities of node *v* across all *j* nodes is equal to 1. The incoming probabilities for node *v*, represented by $f_{jv}$, is the probabilities for random walkers started at other nodes to reach node *v*. In other words, the final random walk probability can be considered as a matrix, where each column has the outgoing probabilities and each row has the incoming probabilities for the corresponding node. The outgoing probabilities always sum to unity. On the other hand, the sum of the probabilities coming from other nodes to a specific node might vary, depending on the degree and topology of the network. The netSmooth uses the incoming probabilities to calculate the imputed value of a gene, whereas our method uses outgoing probabilities. This difference in the approaches indeed has a significant impact on the imputed values that can be best described by what we call the ”hub effect.” The hub effect is defined as large variations in the expression value of a hub gene when incoming probabilities are unrestrictedly used to impute the network. In other words, there exists a high correlation between the degree and imputed value of a given gene. To investigate the impact of different diffusion methods we implemented both approaches and applied them to human brain data. As can be seen in Figure 5, hub nodes are severely affected by imputation when incoming probabilities are used (netSmooth), while the effect is minimum when outgoing probabilities are used (netImpute).

## 4. Conclusions

In this work, we introduced an algorithm to tackle the technical issues of scRNA-sequencing protocols which lead to dropout events appearing as excess zeros in the read counts, a measure of expression level in genes among different cells. The proposed method, netImpute, is a flexible tool capable of utilizing information from different sources including gene co-expression network, cell co-expression network, and protein-protein interaction network. The method was tested on simulated as well as several real datasets covering a wide array of dropout intensity and cell types diversity. Evaluation results showed that netImpute was able to denoise the read count matrix and recover missing transcripts by imputing gene expression profiles via the implementation of a data diffusion scheme. It dramatically enhanced visualization and cell-type identification of zero-inflated scRNA-seq data and outperformed several existing tools. Overall, netImpute demonstrated promising potential to be utilized as a pre-processing tool to denoise scRNA-seq data for downstream analysis such as cell type identification. 

## Figures and Tables

**Table 1:** The scRNA-seq datasets used for benchmarking.

Dataset	Num of Cells	Num of Clusters	Accession No	Ref
Human brain	420	8	GSE67835	[23]
Deng	286	10	GSE45719	[24]
Pollen	300	11	SRP041736	[25]
Usoskin	622	4	GSE102827	[26]
Zeisel	3005	7	GSE60361	[27]
Baron human	8569	14	GSE84133	[28]
Treutlein	80	5	GSE52583	[29]

**Table 2:** Treutlein confusion matrix.

	PCA + Kmeans on Raw Data					SC3 on Raw Data					PCA + Kmeans on Imputed Data					SC3 on Imputed Data				
Cell Type	c1	c2	c3	c4	c5	c1	c2	c3	c4	c5	c1	c2	c3	c4	c5	c1	c2	c3	c4	c5
AT1	18	0	20	3	0	8	0	33	0	0	33	0	0	8	0	41	0	0	0	0
AT2	1	0	0	0	11	0	0	0	0	12	0	12	0	0	0	0	0	12	0	0
BP	1	0	9	0	3	0	7	3	0	3	0	3	0	10	0	1	9	3	0	0
Ciliated	0	3	0	0	0	0	0	0	3	0	0	0	0	0	3	0	0	0	3	0
Clara	0	10	1	0	0	0	1	0	10	0	0	0	10	1	0	0	0	0	0	11

**Table 3:** Run time of netImpute and other imputation methods.

Dataset	netImpute	MAGIC	SAVER-doFast	SAVER	netSmooth	scImpute
Baron (8569 cells)	270 (s)	90 (s)	7396(s)	5 (days)	2640 (s)	29944 (s)
Zeisel (3005 cells)	315 (s)	14 (s)	5104 (s)	4 (days)	1320 (s)	26848 (s)
